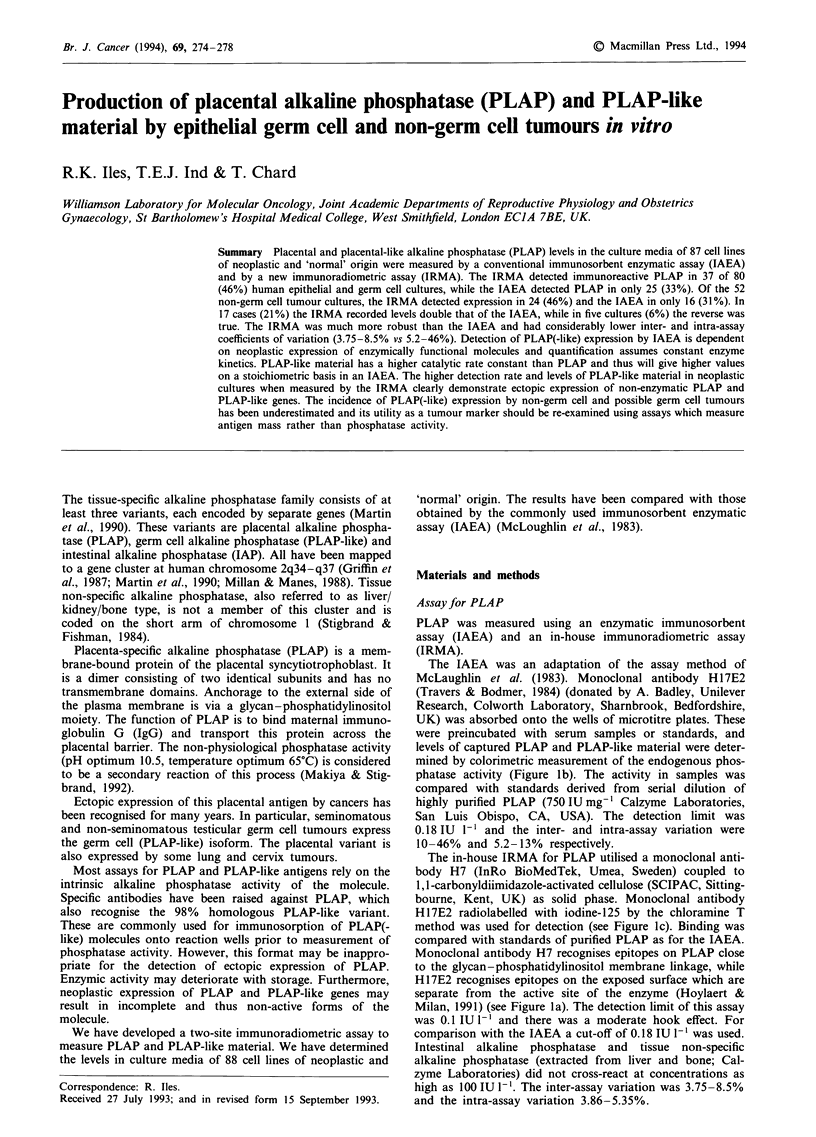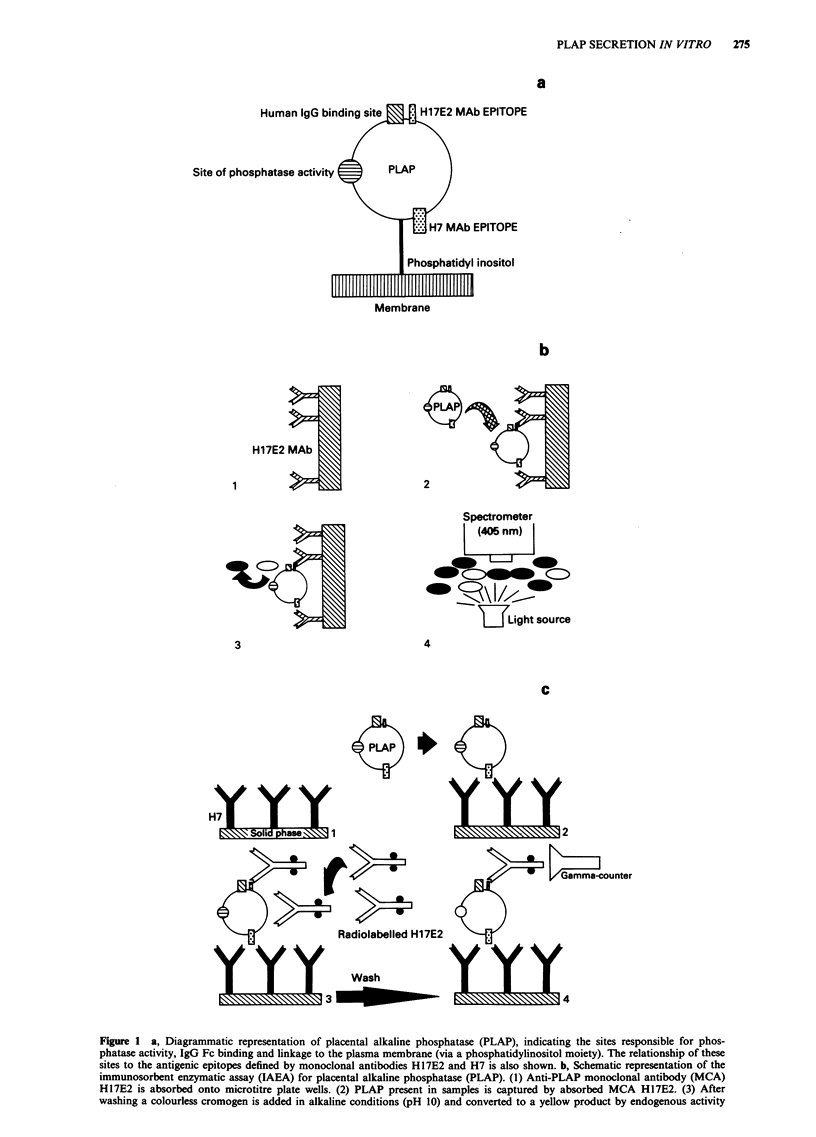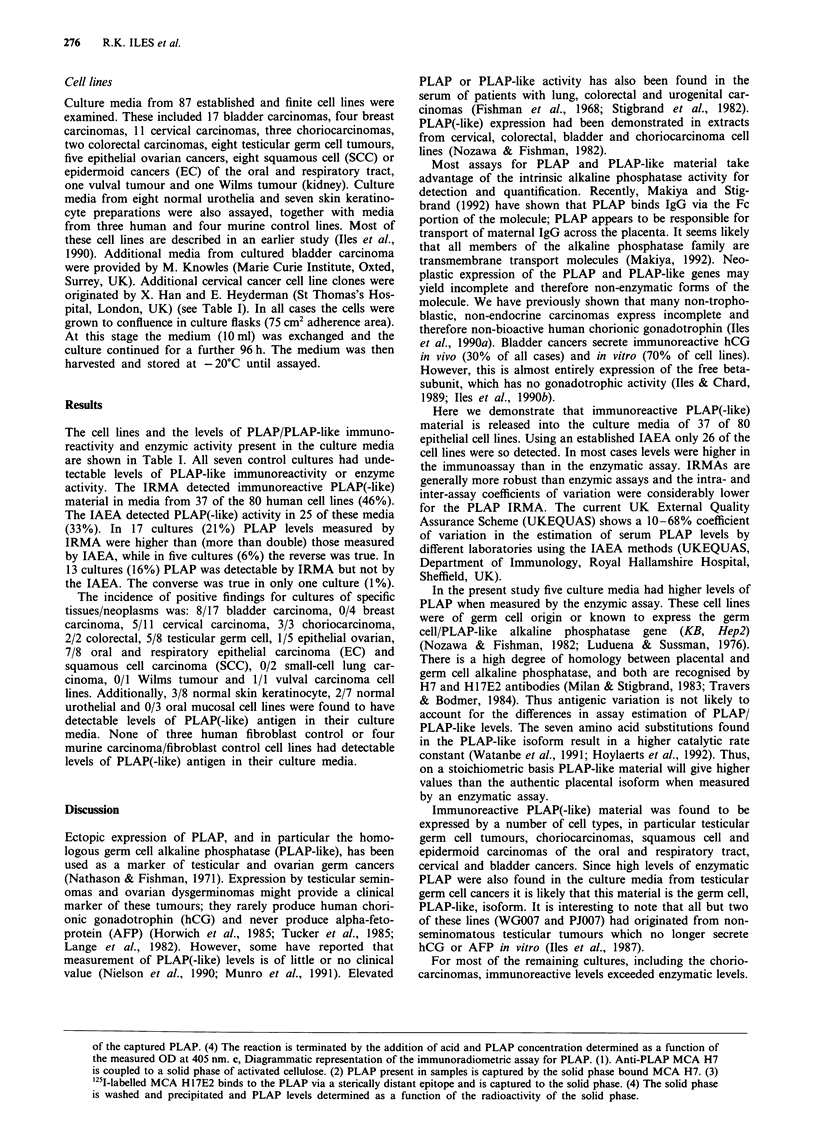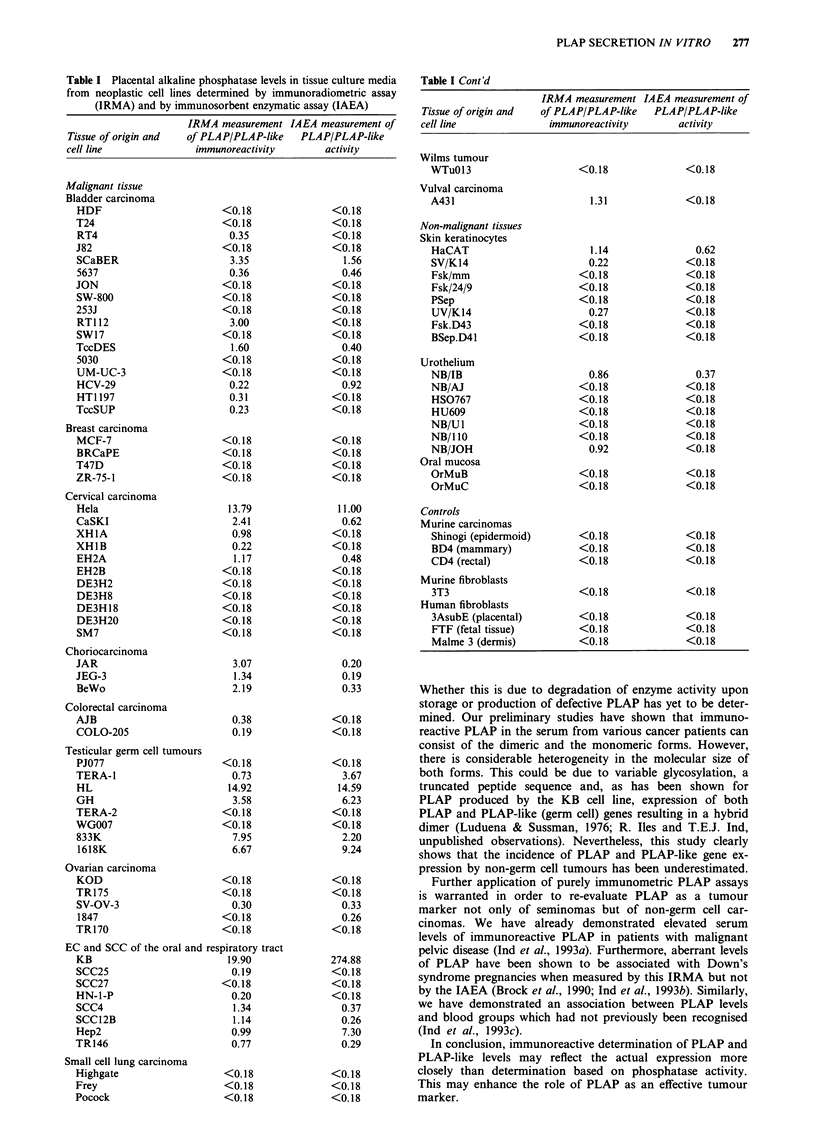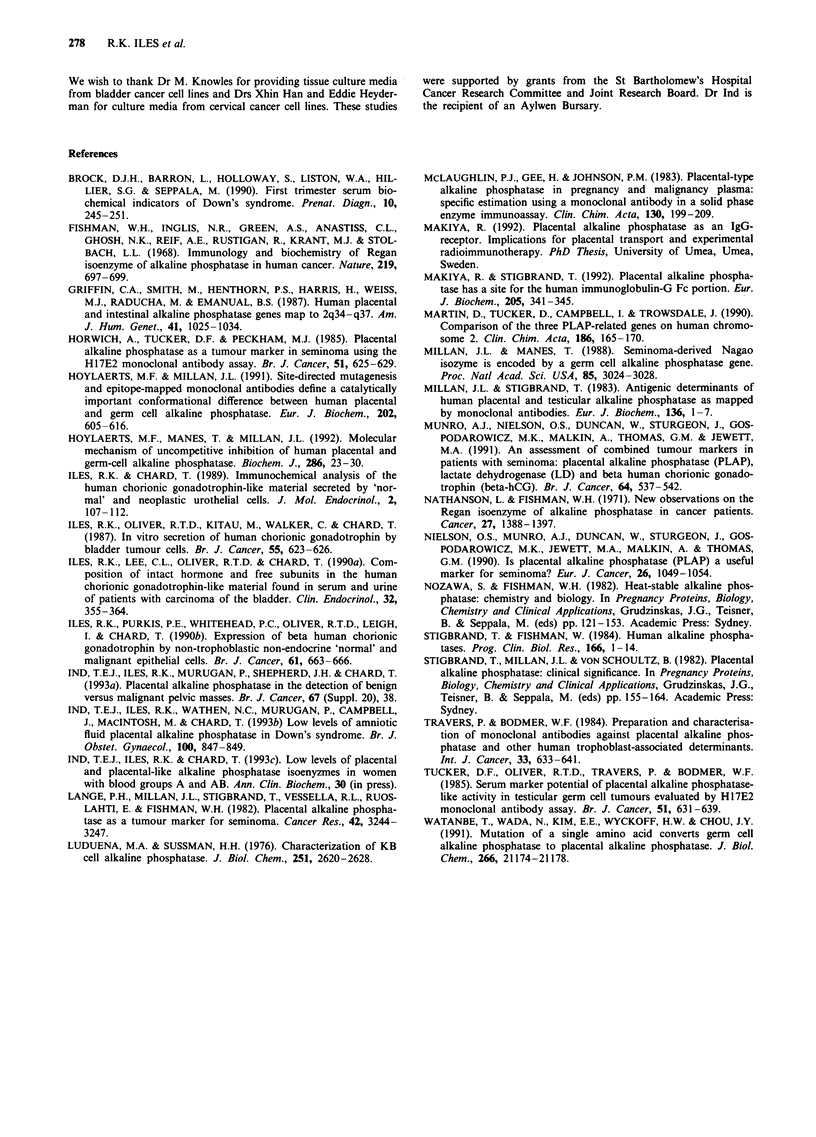# Production of placental alkaline phosphatase (PLAP) and PLAP-like material by epithelial germ cell and non-germ cell tumours in vitro.

**DOI:** 10.1038/bjc.1994.51

**Published:** 1994-02

**Authors:** R. K. Iles, T. E. Ind, T. Chard

**Affiliations:** Williamson Laboratory for Molecular Oncology, Joint Academic Department of Reproductive Physiology, St Bartholomew's Hospital Medical College, West Smithfield, London, UK.

## Abstract

Placental and placental-like alkaline phosphatase (PLAP) levels in the culture media of 87 cell lines of neoplastic and 'normal' origin were measured by a conventional immunosorbent enzymatic assay (IAEA) and by a new immunoradiometric assay (IRMA). The IRMA detected immunoreactive PLAP in 37 of 80 (46%) human epithelial and germ cell cultures, while the IAEA detected PLAP in only 25 (33%). Of the 52 non-germ cell tumour cultures, the IRMA detected expression in 24 (46%) and the IAEA in only 16 (31%). In 17 cases (21%) the IRMA recorded levels double that of the IAEA, while in five cultures (6%) the reverse was true. The IRMA was much more robust than the IAEA and had considerably lower inter- and intra-assay coefficients of variation (3.75-8.5% vs 5.2-46%). Detection of PLAP(-like) expression by IAEA is dependent on neoplastic expression of enzymatically functional molecules and quantification assumes constant enzyme kinetics. PLAP-like material has a higher catalytic rate constant than PLAP and thus will give higher values on a stoichiometric basis in an IAEA. The higher detection rate and levels of PLAP-like material in neoplastic cultures when measured by the IRMA clearly demonstrate ectopic expression of non-enzymatic PLAP and PLAP-like genes. The incidence of PLAP(-like) expression by non-germ cell and possible germ cell tumours has been underestimated and its utility as a tumour marker should be re-examined using assays which measure antigen mass rather than phosphatase activity.


Br. J. Cancer (1994), 69, 274–278 © Macmillan Press Ltd., 1994

### Production of placental alkaline phosphatase (PLAP) and PLAP-like material by epithelial germ cell and non-germ cell tumours *in vitro*

R.K. Iles, T.E.J. Ind & T. Chard

*Williamson Laboratory for Molecular Oncology, Joint Academic Departments of Reproductive Physiology and Obstetrics Gynaecology, St Bartholomew's Hospital Medical College, West Smithfield, London EC1A 7BE, UK.*

**Summary** Placental and placental-like alkaline phosphatase (PLAP) levels in the culture media of 87 cell lines of neoplastic and 'normal' origin were measured by a conventional immunosorbent enzymatic assay (IAEA) and by a new immunoradiometric assay (IRMA). The IRMA detected immunoreactive PLAP in 37 of 80 (46%) human epithelial and germ cell cultures, while the IAEA detected PLAP in only 25 (33%). Of the 52 non-germ cell tumour cultures, the IRMA detected expression in 24 (46%) and the IAEA in only 16 (31%). In 17 cases (21%) the IRMA recorded levels double that of the IAEA, while in five cultures (6%) the reverse was true. The IRMA was much more robust than the IAEA and had considerably lower inter- and intra-assay coefficients of variation (3.75–8.5% *vs* 5.2–46%). Detection of PLAP(-like) expression by IAEA is dependent on neoplastic expression of enzymically functional molecules and quantification assumes constant enzyme kinetics. PLAP-like material has a higher catalytic rate constant than PLAP and thus will give higher values on a stoichiometric basis in an IAEA. The higher detection rate and levels of PLAP-like material in neoplastic cultures when measured by the IRMA clearly demonstrate ectopic expression of non-enzymatic PLAP and PLAP-like genes. The incidence of PLAP(-like) expression by non-germ cell and possible germ cell tumours has been underestimated and its utility as a tumour marker should be re-examined using assays which measure antigen mass rather than phosphatase activity.

The tissue-specific alkaline phosphatase family consists of at least three variants, each encoded by separate genes (Martin *et al.*, 1990). These variants are placental alkaline phosphatase (PLAP), germ cell alkaline phosphatase (PLAP-like) and intestinal alkaline phosphatase (IAP). All have been mapped to a gene cluster at human chromosome 2q34–q37 (Griffin *et al.*, 1987; Martin *et al.*, 1990; Millan & Manes, 1988). Tissue non-specific alkaline phosphatase, also referred to as liver/kidney/bone type, is not a member of this cluster and is coded on the short arm of chromosome 1 (Stigbrand & Fishman, 1984).

Placenta-specific alkaline phosphatase (PLAP) is a membrane-bound protein of the placental syncytiotrophoblast. It is a dimer consisting of two identical subunits and has no transmembrane domains. Anchorage to the external side of the plasma membrane is via a glycan–phosphatidylinositol moiety. The function of PLAP is to bind maternal immunoglobulin G (IgG) and transport this protein across the placental barrier. The non-physiological phosphatase activity (pH optimum 10.5, temperature optimum 65°C) is considered to be a secondary reaction of this process (Makiya & Stigbrand, 1992).

Ectopic expression of this placental antigen by cancers has been recognised for many years. In particular, seminomatous and non-seminomatous testicular germ cell tumours express the germ cell (PLAP-like) isoform. The placental variant is also expressed by some lung and cervix tumours.

Most assays for PLAP and PLAP-like antigens rely on the intrinsic alkaline phosphatase activity of the molecule. Specific antibodies have been raised against PLAP, which also recognise the 98% homologous PLAP-like variant. These are commonly used for immunosorption of PLAP(-like) molecules onto reaction wells prior to measurement of phosphatase activity. However, this format may be inappropriate for the detection of ectopic expression of PLAP. Enzymic activity may deteriorate with storage. Furthermore, neoplastic expression of PLAP and PLAP-like genes may result in incomplete and thus non-active forms of the molecule.

We have developed a two-site immunoradiometric assay to measure PLAP and PLAP-like material. We have determined the levels in culture media of 88 cell lines of neoplastic and 'normal' origin. The results have been compared with those obtained by the commonly used immunosorbent enzymatic assay (IAEA) (McLoughlin *et al.*, 1983).

Correspondence: R. Iles.
Received 27 July 1993; and in revised form 15 September 1993.

### Materials and methods

### *Assay for PLAP*

PLAP was measured using an enzymatic immunosorbent assay (IAEA) and an in-house immunoradiometric assay (IRMA).

The IAEA was an adaptation of the assay method of McLaughlin *et al.* (1983). Monoclonal antibody H17E2 (Travers & Bodmer, 1984) (donated by A. Badley, Unilever Research, Colworth Laboratory, Sharnbrook, Bedfordshire, UK) was absorbed onto the wells of microtitre plates. These were preincubated with serum samples or standards, and levels of captured PLAP and PLAP-like material were determined by colorimetric measurement of the endogenous phosphatase activity (Figure 1b). The activity in samples was compared with standards derived from serial dilution of highly purified PLAP (750 IU mg$^{-1}$ Calzyme Laboratories, San Luis Obispo, CA, USA). The detection limit was 0.18 IU l$^{-1}$ and the inter- and intra-assay variation were 10–46% and 5.2–13% respectively.

The in-house IRMA for PLAP utilised a monoclonal antibody H7 (InRo BioMedTek, Umea, Sweden) coupled to 1,1-carbonyldiimidazole-activated cellulose (SCIPAC, Sittingbourne, Kent, UK) as solid phase. Monoclonal antibody H17E2 radiolabelled with iodine-125 by the chloramine T method was used for detection (see Figure 1c). Binding was compared with standards of purified PLAP as for the IAEA. Monoclonal antibody H7 recognises epitopes on PLAP close to the glycan–phosphatidylinositol membrane linkage, while H17E2 recognises epitopes on the exposed surface which are separate from the active site of the enzyme (Hoylaert & Milan, 1991) (see Figure 1a). The detection limit of this assay was 0.1 IU l$^{-1}$ and there was a moderate hook effect. For comparison with the IAEA a cut-off of 0.18 IU l$^{-1}$ was used. Intestinal alkaline phosphatase and tissue non-specific alkaline phosphatase (extracted from liver and bone; Calzyme Laboratories) did not cross-react at concentrations as high as 100 IU l$^{-1}$. The inter-assay variation was 3.75–8.5% and the intra-assay variation 3.86–5.35%.

**Figure 1** **a**, Diagrammatic representation of placental alkaline phosphatase (PLAP), indicating the sites responsible for phosphatase activity, IgG Fc binding and linkage to the plasma membrane (via a phosphatidylinositol moiety). The relationship of these sites to the antigenic epitopes defined by monoclonal antibodies H17E2 and H7 is also shown. **b**, Schematic representation of the immunosorbent enzymatic assay (IAEA) for placental alkaline phosphatase (PLAP). (1) Anti-PLAP monoclonal antibody (MCA) H17E2 is absorbed onto microtitre plate wells. (2) PLAP present in samples is captured by absorbed MCA H17E2. (3) After washing a colourless cromogen is added in alkaline conditions (pH 10) and converted to a yellow product by endogenous activity

*Cell lines*

Culture media from 87 established and finite cell lines were examined. These included 17 bladder carcinomas, four breast carcinomas, 11 cervical carcinomas, three choriocarcinomas, two colorectal carcinomas, eight testicular germ cell tumours, five epithelial ovarian cancers, eight squamous cell (SCC) or epidermoid cancers (EC) of the oral and respiratory tract, one vulval tumour and one Wilms tumour (kidney). Culture media from eight normal urothelia and seven skin keratinocyte preparations were also assayed, together with media from three human and four murine control lines. Most of these cell lines are described in an earlier study (Iles *et al.*, 1990). Additional media from cultured bladder carcinoma were provided by M. Knowles (Marie Curie Institute, Oxted, Surrey, UK). Additional cervical cancer cell line clones were originated by X. Han and E. Heyderman (St Thomas's Hospital, London, UK) (see Table I). In all cases the cells were grown to confluence in culture flasks (75 $cm^2$ adherence area). At this stage the medium (10 ml) was exchanged and the culture continued for a further 96 h. The medium was then harvested and stored at $-20°C$ until assayed.

### Results

The cell lines and the levels of PLAP/PLAP-like immunoreactivity and enzymic activity present in the culture media are shown in Table I. All seven control cultures had undetectable levels of PLAP-like immunoreactivity or enzyme activity. The IRMA detected immunoreactive PLAP(-like) material in media from 37 of the 80 human cell lines (46%). The IAEA detected PLAP(-like) activity in 25 of these media (33%). In 17 cultures (21%) PLAP levels measured by IRMA were higher than (more than double) those measured by IAEA, while in five cultures (6%) the reverse was true. In 13 cultures (16%) PLAP was detectable by IRMA but not by the IAEA. The converse was true in only one culture (1%).

The incidence of positive findings for cultures of specific tissues/neoplasms was: 8/17 bladder carcinoma, 0/4 breast carcinoma, 5/11 cervical carcinoma, 3/3 choriocarcinoma, 2/2 colorectal, 5/8 testicular germ cell, 1/5 epithelial ovarian, 7/8 oral and respiratory epithelial carcinoma (EC) and squamous cell carcinoma (SCC), 0/2 small-cell lung carcinoma, 0/1 Wilms tumour and 1/1 vulval carcinoma cell lines. Additionally, 3/8 normal skin keratinocyte, 2/7 normal urothelial and 0/3 oral mucosal cell lines were found to have detectable levels of PLAP(-like) antigen in their culture media. None of three human fibroblast control or four murine carcinoma/fibroblast control cell lines had detectable levels of PLAP(-like) antigen in their culture media.

### Discussion

Ectopic expression of PLAP, and in particular the homologous germ cell alkaline phosphatase (PLAP-like), has been used as a marker of testicular and ovarian germ cancers (Nathason & Fishman, 1971). Expression by testicular seminomas and ovarian dysgerminomas might provide a clinical marker of these tumours; they rarely produce human chorionic gonadotrophin (hCG) and never produce alpha-fetoprotein (AFP) (Horwich *et al.*, 1985; Tucker *et al.*, 1985; Lange *et al.*, 1982). However, some have reported that measurement of PLAP(-like) levels is of little or no clinical value (Nielson *et al.*, 1990; Munro *et al.*, 1991). Elevated PLAP or PLAP-like activity has also been found in the serum of patients with lung, colorectal and urogenital carcinomas (Fishman *et al.*, 1968; Stigbrand *et al.*, 1982). PLAP(-like) expression had been demonstrated in extracts from cervical, colorectal, bladder and choriocarcinoma cell lines (Nozawa & Fishman, 1982).

Most assays for PLAP and PLAP-like material take advantage of the intrinsic alkaline phosphatase activity for detection and quantification. Recently, Makiya and Stigbrand (1992) have shown that PLAP binds IgG via the Fc portion of the molecule; PLAP appears to be responsible for transport of maternal IgG across the placenta. It seems likely that all members of the alkaline phosphatase family are transmembrane transport molecules (Makiya, 1992). Neoplastic expression of the PLAP and PLAP-like genes may yield incomplete and therefore non-enzymatic forms of the molecule. We have previously shown that many non-trophoblastic, non-endocrine carcinomas express incomplete and therefore non-bioactive human chorionic gonadotrophin (Iles *et al.*, 1990*a*). Bladder cancers secrete immunoreactive hCG *in vivo* (30% of all cases) and *in vitro* (70% of cell lines). However, this is almost entirely expression of the free beta-subunit, which has no gonadotrophic activity (Iles & Chard, 1989; Iles *et al.*, 1990*b*).

Here we demonstrate that immunoreactive PLAP(-like) material is released into the culture media of 37 of 80 epithelial cell lines. Using an established IAEA only 26 of the cell lines were so detected. In most cases levels were higher in the immunoassay than in the enzymatic assay. IRMAs are generally more robust than enzymic assays and the intra- and inter-assay coefficients of variation were considerably lower for the PLAP IRMA. The current UK External Quality Assurance Scheme (UKEQUAS) shows a 10–68% coefficient of variation in the estimation of serum PLAP levels by different laboratories using the IAEA methods (UKEQUAS, Department of Immunology, Royal Hallamshire Hospital, Sheffield, UK).

In the present study five culture media had higher levels of PLAP when measured by the enzymic assay. These cell lines were of germ cell origin or known to express the germ cell/PLAP-like alkaline phosphatase gene (*KB*, *Hep2*) (Nozawa & Fishman, 1982; Luduena & Sussman, 1976). There is a high degree of homology between placental and germ cell alkaline phosphatase, and both are recognised by H7 and H17E2 antibodies (Milan & Stigbrand, 1983; Travers & Bodmer, 1984). Thus antigenic variation is not likely to account for the differences in assay estimation of PLAP/PLAP-like levels. The seven amino acid substitutions found in the PLAP-like isoform result in a higher catalytic rate constant (Watanbe *et al.*, 1991; Hoylaerts *et al.*, 1992). Thus, on a stoichiometric basis PLAP-like material will give higher values than the authentic placental isoform when measured by an enzymatic assay.

Immunoreactive PLAP(-like) material was found to be expressed by a number of cell types, in particular testicular germ cell tumours, choriocarcinomas, squamous cell and epidermoid carcinomas of the oral and respiratory tract, cervical and bladder cancers. Since high levels of enzymatic PLAP were also found in the culture media from testicular germ cell cancers it is likely that this material is the germ cell, PLAP-like, isoform. It is interesting to note that all but two of these lines (WG007 and PJ007) had originated from non-seminomatous testicular tumours which no longer secrete hCG or AFP *in vitro* (Iles *et al.*, 1987).

For most of the remaining cultures, including the choriocarcinomas, immunoreactive levels exceeded enzymatic levels.

of the captured PLAP. (4) The reaction is terminated by the addition of acid and PLAP concentration determined as a function of the measured OD at 405 nm. **c**, Diagrammatic representation of the immunoradiometric assay for PLAP. (1). Anti-PLAP MCA H7 is coupled to a solid phase of activated cellulose. (2) PLAP present in samples is captured by the solid phase bound MCA H7. (3) $^{125}I$-labelled MCA H17E2 binds to the PLAP via a sterically distant epitope and is captured to the solid phase. (4) The solid phase is washed and precipitated and PLAP levels determined as a function of the radioactivity of the solid phase.

**Table I** Placental alkaline phosphatase levels in tissue culture media from neoplastic cell lines determined by immunoradiometric assay (IRMA) and by immunosorbent enzymatic assay (IAEA)

| *Tissue of origin and cell line* | *IRMA measurement of PLAP/PLAP-like immunoreactivity* | *IAEA measurement of PLAP/PLAP-like activity* |
|---|---|---|
| *Malignant tissue* | | |
| Bladder carcinoma | | |
| HDF | <0.18 | <0.18 |
| T24 | <0.18 | <0.18 |
| RT4 | 0.35 | <0.18 |
| J82 | <0.18 | <0.18 |
| SCaBER | 3.35 | 1.56 |
| 5637 | 0.36 | 0.46 |
| JON | <0.18 | <0.18 |
| SW-800 | <0.18 | <0.18 |
| 253J | <0.18 | <0.18 |
| RT112 | 3.00 | <0.18 |
| SW17 | <0.18 | <0.18 |
| TccDES | 1.60 | 0.40 |
| 5030 | <0.18 | <0.18 |
| UM-UC-3 | <0.18 | <0.18 |
| HCV-29 | 0.22 | 0.92 |
| HT1197 | 0.31 | <0.18 |
| TccSUP | 0.23 | <0.18 |
| Breast carcinoma | | |
| MCF-7 | <0.18 | <0.18 |
| BRCaPE | <0.18 | <0.18 |
| T47D | <0.18 | <0.18 |
| ZR-75-1 | <0.18 | <0.18 |
| Cervical carcinoma | | |
| Hela | 13.79 | 11.00 |
| CaSKI | 2.41 | 0.62 |
| XH1A | 0.98 | <0.18 |
| XH1B | 0.22 | <0.18 |
| EH2A | 1.17 | 0.48 |
| EH2B | <0.18 | <0.18 |
| DE3H2 | <0.18 | <0.18 |
| DE3H8 | <0.18 | <0.18 |
| DE3H18 | <0.18 | <0.18 |
| DE3H20 | <0.18 | <0.18 |
| SM7 | <0.18 | <0.18 |
| Choriocarcinoma | | |
| JAR | 3.07 | 0.20 |
| JEG-3 | 1.34 | 0.19 |
| BeWo | 2.19 | 0.33 |
| Colorectal carcinoma | | |
| AJB | 0.38 | <0.18 |
| COLO-205 | 0.19 | <0.18 |
| Testicular germ cell tumours | | |
| PJ077 | <0.18 | <0.18 |
| TERA-1 | 0.73 | 3.67 |
| HL | 14.92 | 14.59 |
| GH | 3.58 | 6.23 |
| TERA-2 | <0.18 | <0.18 |
| WG007 | <0.18 | <0.18 |
| 833K | 7.95 | 2.20 |
| 1618K | 6.67 | 9.24 |
| Ovarian carcinoma | | |
| KOD | <0.18 | <0.18 |
| TR175 | <0.18 | <0.18 |
| SV-OV-3 | 0.30 | 0.33 |
| 1847 | <0.18 | 0.26 |
| TR170 | <0.18 | <0.18 |
| EC and SCC of the oral and respiratory tract | | |
| KB | 19.90 | 274.88 |
| SCC25 | 0.19 | <0.18 |
| SCC27 | <0.18 | <0.18 |
| HN-1-P | 0.20 | <0.18 |
| SCC4 | 1.34 | 0.37 |
| SCC12B | 1.14 | 0.26 |
| Hep2 | 0.99 | 7.30 |
| TR146 | 0.77 | 0.29 |
| Small cell lung carcinoma | | |
| Highgate | <0.18 | <0.18 |
| Frey | <0.18 | <0.18 |
| Pocock | <0.18 | <0.18 |

**Table I** *Cont'd*

| *Tissue of origin and cell line* | *IRMA measurement of PLAP/PLAP-like immunoreactivity* | *IAEA measurement of PLAP/PLAP-like activity* |
|---|---|---|
| Wilms tumour | | |
| WTu013 | <0.18 | <0.18 |
| Vulval carcinoma | | |
| A431 | 1.31 | <0.18 |
| *Non-malignant tissues* | | |
| Skin keratinocytes | | |
| HaCAT | 1.14 | 0.62 |
| SV/K14 | 0.22 | <0.18 |
| Fsk/mm | <0.18 | <0.18 |
| Fsk/24/9 | <0.18 | <0.18 |
| PSep | <0.18 | <0.18 |
| UV/K14 | 0.27 | <0.18 |
| Fsk.D43 | <0.18 | <0.18 |
| BSep.D41 | <0.18 | <0.18 |
| Urothelium | | |
| NB/IB | 0.86 | 0.37 |
| NB/AJ | <0.18 | <0.18 |
| HSO767 | <0.18 | <0.18 |
| HU609 | <0.18 | <0.18 |
| NB/U1 | <0.18 | <0.18 |
| NB/110 | <0.18 | <0.18 |
| NB/JOH | 0.92 | <0.18 |
| Oral mucosa | | |
| OrMuB | <0.18 | <0.18 |
| OrMuC | <0.18 | <0.18 |
| *Controls* | | |
| Murine carcinomas | | |
| Shinogi (epidermoid) | <0.18 | <0.18 |
| BD4 (mammary) | <0.18 | <0.18 |
| CD4 (rectal) | <0.18 | <0.18 |
| Murine fibroblasts | | |
| 3T3 | <0.18 | <0.18 |
| Human fibroblasts | | |
| 3AsubE (placental) | <0.18 | <0.18 |
| FTF (fetal tissue) | <0.18 | <0.18 |
| Malme 3 (dermis) | <0.18 | <0.18 |

Whether this is due to degradation of enzyme activity upon storage or production of defective PLAP has yet to be determined. Our preliminary studies have shown that immunoreactive PLAP in the serum from various cancer patients can consist of the dimeric and the monomeric forms. However, there is considerable heterogeneity in the molecular size of both forms. This could be due to variable glycosylation, a truncated peptide sequence and, as has been shown for PLAP produced by the KB cell line, expression of both PLAP and PLAP-like (germ cell) genes resulting in a hybrid dimer (Luduena & Sussman, 1976; R. Iles and T.E.J. Ind, unpublished observations). Nevertheless, this study clearly shows that the incidence of PLAP and PLAP-like gene expression by non-germ cell tumours has been underestimated.

Further application of purely immunometric PLAP assays is warranted in order to re-evaluate PLAP as a tumour marker not only of seminomas but of non-germ cell carcinomas. We have already demonstrated elevated serum levels of immunoreactive PLAP in patients with malignant pelvic disease (Ind *et al.*, 1993*a*). Furthermore, aberrant levels of PLAP have been shown to be associated with Down's syndrome pregnancies when measured by this IRMA but not by the IAEA (Brock *et al.*, 1990; Ind *et al.*, 1993*b*). Similarly, we have demonstrated an association between PLAP levels and blood groups which had not previously been recognised (Ind *et al.*, 1993*c*).

In conclusion, immunoreactive determination of PLAP and PLAP-like levels may reflect the actual expression more closely than determination based on phosphatase activity. This may enhance the role of PLAP as an effective tumour marker.

We wish to thank Dr M. Knowles for providing tissue culture media from bladder cancer cell lines and Drs Xhin Han and Eddie Heyderman for culture media from cervical cancer cell lines. These studies were supported by grants from the St Bartholomew's Hospital Cancer Research Committee and Joint Research Board. Dr Ind is the recipient of an Aylwen Bursary.

### References

BROCK, D.J.H., BARRON, L., HOLLOWAY, S., LISTON, W.A., HILLIER, S.G. & SEPPALA, M. (1990). First trimester serum biochemical indicators of Down's syndrome. *Prenat. Diagn.*, **10**, 245–251.

FISHMAN, W.H., INGLIS, N.R., GREEN, A.S., ANASTISS, C.L., GHOSH, N.K., REIF, A.E., RUSTIGAN, R., KRANT, M.J. & STOLBACH, L.L. (1968). Immunology and biochemistry of Regan isoenzyme of alkaline phosphatase in human cancer. *Nature*, **219**, 697–699.

GRIFFIN, C.A., SMITH, M., HENTHORN, P.S., HARRIS, H., WEISS, M.J., RADUCHA, M. & EMANUAL, B.S. (1987). Human placental and intestinal alkaline phosphatase genes map to 2q34–q37. *Am. J. Hum. Genet.*, **41**, 1025–1034.

HORWICH, A., TUCKER, D.F. & PECKHAM, M.J. (1985). Placental alkaline phosphatase as a tumour marker in seminoma using the H17E2 monoclonal antibody assay. *Br. J. Cancer*, **51**, 625–629.

HOYLAERTS, M.F. & MILLAN, J.L. (1991). Site-directed mutagenesis and epitope-mapped monoclonal antibodies define a catalytically important conformational difference between human placental and germ cell alkaline phosphatase. *Eur. J. Biochem.*, **202**, 605–616.

HOYLAERTS, M.F., MANES, T. & MILLAN, J.L. (1992). Molecular mechanism of uncompetitive inhibition of human placental and germ-cell alkaline phosphatase. *Biochem. J.*, **286**, 23–30.

ILES, R.K. & CHARD, T. (1989). Immunochemical analysis of the human chorionic gonadotrophin-like material secreted by 'normal' and neoplastic urothelial cells. *J. Mol. Endocrinol.*, **2**, 107–112.

ILES, R.K., OLIVER, R.T.D., KITAU, M., WALKER, C. & CHARD, T. (1987). In vitro secretion of human chorionic gonadotrophin by bladder tumour cells. *Br. J. Cancer*, **55**, 623–626.

ILES, R.K., LEE, C.L., OLIVER, R.T.D. & CHARD, T. (1990*a*). Composition of intact hormone and free subunits in the human chorionic gonadotrophin-like material found in serum and urine of patients with carcinoma of the bladder. *Clin. Endocrinol.*, **32**, 355–364.

ILES, R.K., PURKIS, P.E., WHITEHEAD, P.C., OLIVER, R.T.D., LEIGH, I. & CHARD, T. (1990*b*). Expression of beta human chorionic gonadotrophin by non-trophoblastic non-endocrine 'normal' and malignant epithelial cells. *Br. J. Cancer*, **61**, 663–666.

IND, T.E.J., ILES, R.K., MURUGAN, P., SHEPHERD, J.H. & CHARD, T. (1993*a*). Placental alkaline phosphatase in the detection of benign versus malignant pelvic masses. *Br. J. Cancer*, **67** (Suppl. 20), 38.

IND, T.E.J., ILES, R.K., WATHEN, N.C., MURUGAN, P., CAMPBELL, J., MACINTOSH, M. & CHARD, T. (1993*b*) Low levels of amniotic fluid placental alkaline phosphatase in Down's syndrome. *Br. J. Obstet. Gynaecol.*, **100**, 847–849.

IND, T.E.J., ILES, R.K. & CHARD, T. (1993*c*). Low levels of placental and placental-like alkaline phosphatase isoenyzmes in women with blood groups A and AB. *Ann. Clin. Biochem.*, **30** (in press).

LANGE, P.H., MILLAN, J.L., STIGBRAND, T., VESSELLA, R.L., RUOSLAHTI, E. & FISHMAN, W.H. (1982). Placental alkaline phosphatase as a tumour marker for seminoma. *Cancer Res.*, **42**, 3244–3247.

LUDUENA, M.A. & SUSSMAN, H.H. (1976). Characterization of KB cell alkaline phosphatase. *J. Biol. Chem.*, **251**, 2620–2628.

McLAUGHLIN, P.J., GEE, H. & JOHNSON, P.M. (1983). Placental-type alkaline phosphatase in pregnancy and malignancy plasma: specific estimation using a monoclonal antibody in a solid phase enzyme immunoassay. *Clin. Chim. Acta*, **130**, 199–209.

MAKIYA, R. (1992). Placental alkaline phosphatase as an IgG-receptor. Implications for placental transport and experimental radioimmunotherapy. *PhD Thesis*, University of Umea, Umea, Sweden.

MAKIYA, R. & STIGBRAND, T. (1992). Placental alkaline phosphatase has a site for the human immunoglobulin-G Fc portion. *Eur. J. Biochem.*, **205**, 341–345.

MARTIN, D., TUCKER, D., CAMPBELL, I. & TROWSDALE, J. (1990). Comparison of the three PLAP-related genes on human chromosome 2. *Clin. Chim. Acta*, **186**, 165–170.

MILLAN, J.L. & MANES, T. (1988). Seminoma-derived Nagao isozyme is encoded by a germ cell alkaline phosphatase gene. *Proc. Natl Acad. Sci. USA*, **85**, 3024–3028.

MILLAN, J.L. & STIGBRAND, T. (1983). Antigenic determinants of human placental and testicular alkaline phosphatase as mapped by monoclonal antibodies. *Eur. J. Biochem.*, **136**, 1–7.

MUNRO, A.J., NIELSON, O.S., DUNCAN, W., STURGEON, J., GOSPODAROWICZ, M.K., MALKIN, A., THOMAS, G.M. & JEWETT, M.A. (1991). An assessment of combined tumour markers in patients with seminoma: placental alkaline phosphatase (PLAP), lactate dehydrogenase (LD) and beta human chorionic gonadotrophin (beta-hCG). *Br. J. Cancer*, **64**, 537–542.

NATHANSON, L. & FISHMAN, W.H. (1971). New observations on the Regan isoenzyme of alkaline phosphatase in cancer patients. *Cancer*, **27**, 1388–1397.

NIELSON, O.S., MUNRO, A.J., DUNCAN, W., STURGEON, J., GOSPODAROWICZ, M.K., JEWETT, M.A., MALKIN, A. & THOMAS, G.M. (1990). Is placental alkaline phosphatase (PLAP) a useful marker for seminoma? *Eur. J. Cancer*, **26**, 1049–1054.

NOZAWA, S. & FISHMAN, W.H. (1982). Heat-stable alkaline phosphatase: chemistry and biology. In *Pregnancy Proteins, Biology, Chemistry and Clinical Applications*, Grudzinskas, J.G., Teisner, B. & Seppala, M. (eds) pp. 121–153. Academic Press: Sydney.

STIGBRAND, T. & FISHMAN, W. (1984). Human alkaline phosphatases. *Prog. Clin. Biol. Res.*, **166**, 1–14.

STIGBRAND, T., MILLAN, J.L. & VON SCHOULTZ, B. (1982). Placental alkaline phosphatase: clinical significance. In *Pregnancy Proteins, Biology, Chemistry and Clinical Applications*, Grudzinskas, J.G., Teisner, B. & Seppala, M. (eds) pp. 155–164. Academic Press: Sydney.

TRAVERS, P. & BODMER, W.F. (1984). Preparation and characterisation of monoclonal antibodies against placental alkaline phosphatase and other human trophoblast-associated determinants. *Int. J. Cancer*, **33**, 633–641.

TUCKER, D.F., OLIVER, R.T.D., TRAVERS, P. & BODMER, W.F. (1985). Serum marker potential of placental alkaline phosphatase-like activity in testicular germ cell tumours evaluated by H17E2 monoclonal antibody assay. *Br. J. Cancer*, **51**, 631–639.

WATANBE, T., WADA, N., KIM, E.E., WYCKOFF, H.W. & CHOU, J.Y. (1991). Mutation of a single amino acid converts germ cell alkaline phosphatase to placental alkaline phosphatase. *J. Biol. Chem.*, **266**, 21174–21178.